# Time in Service and Resilience in Active Military Personnel during the COVID-19 Pandemic: A Cross-Sectional Study in Northern Peru

**DOI:** 10.3390/ijerph191711052

**Published:** 2022-09-03

**Authors:** Mario J. Valladares-Garrido, Yanela Huamani-Colquichagua, Claudia Anchay-Zuloeta, Cinthia K. Picón-Reátegui, Danai Valladares-Garrido

**Affiliations:** 1 South American Center for Education and Research in Public Health, Universidad Norbert Wiener, Lima 15046, Peru; 2 Oficina de Epidemiología, Hospital Regional Lambayeque, Chiclayo 14012, Peru; 3 Facultad de Medicina Hipólito Unanue, Universidad Nacional Federico Villarreal, Lima 15088, Peru; 4 Facultad de Medicina, Universidad de San Martín de Porres, Chiclayo 14012, Peru; 5 Sociedad Científica de Estudiantes de Medicina Veritas (SCIEMVE), Chiclayo 14012, Peru; 6 Facultad de Medicina, Universidad Cesar Vallejo, Piura 22700, Peru

**Keywords:** resilience, military, COVID-19, coping strategies, Peru

## Abstract

Greater occupational exposure may have a positive effect on the development of resilience. We aimed to determine the association between working time and resilience in Peruvian military personnel during the COVID-19 pandemic. A secondary data analysis was performed including 586 records of military personnel who supported the health emergency during the second epidemic wave in Lambayeque, Peru. Resilience was measured with the short form of the Connor-Davidson Resilience Scale (CD-RISC). Working time and other relevant covariates were collected by self-report. Generalized linear models were used. The mean resilience score was 22.18 and 43.2% scored high for resilience. Participants reported that they are strong individuals when facing difficulties (42.3%), are able to handle unpleasant feelings (40.3%), and achieve their goals despite obstacles (40.4%). Working more than 18 months was associated with a 35% higher prevalence of high resilience (PR: 1.35; 95% CI: 1.05–1.75). In conclusion, a notable number of military personnel experienced high levels of resilience during the pandemic. Working time may have played an important role in the development of this ability. Our findings could help guide the deployment and organization of the military in health emergency support missions.

## 1. Introduction

Resilience can be defined as the “ability to maintain one’s orientation towards existential purposes despite enduring adversities and stressful events” [1]. This concept has arisen from multiple perspectives that have been linked to the (1) ability to recover, (2) type of functioning that characterizes the individual, (3) capacity to bounce back, (4) dynamic process evolving over time, and (5) positive adaptation to life conditions. This capacity is conditioned by certain individual and environmental factors, which come together to produce an exceptional reaction to a major threat [2]. This is why this term has been relevant during the COVID-19 pandemic, given that it has significantly impacted the mental health and academic and work performance of students [3,4], the general population [5,6,7], health personnel [8,9], and front-line military defense personnel [10].

Previous studies have estimated a high level of resilience in health personnel, military, students, and the general population in the context of COVID-19 [11,12,13,14,15]. In a study conducted in the U.S. during the COVID-19 confinement, high levels of resilience were found using the Connor-Davidson Resilience Scale (CD-RISC) [12]. In health personnel, a high level of resilience was found in physicians, nursing technicians, and obstetricians [11]. Similar positive attitudes towards the pandemic have been reported in several studies among university students in Jordania [16], China [17], Pakistan [18], and South Africa [19]. Considering military personnel, a national study of U.S. military veterans found that two-thirds of military personnel exposed to pandemic stressors exhibited a pattern of resilience [13], while a study of U.S. Air Force personnel found a high mean resilience score [20]. Some pre-pandemic studies in this population were also reported. A U.S. study of active military personnel found a high resilience pattern [21] and another of U.S. veterans showed that 75% of military personnel had a high level of resilience [22]. A third study of veterans from the same country showed that the mean resilience score was over the 50th percentile [23]. In the Irish military, a moderate level of resilience was found [24], and in the Swiss population, a study found a mean resilience score close to the 75th percentile [25]. In Colombia, it was observed that military personnel showed low levels of resilience [26], and in Peru, a study of the VRAEM military showed a resilience frequency of 60% [27]. However, these studies used different measures of resilience and it is difficult to make adequate comparisons.

Factors influencing resilience in the general population are increased daily exercise, increased family, partner and social support, increased frequency of prayer, and reduced severity of insomnia symptoms [12]. However, as far as we know, there are no studies on resilience amongst the military personnel during the pandemic, and those in other populations [28,29,30,31,32] have little-explored factors such as religion (related to spiritual support), alcoholism/smoking (related to psychological distress), and history of mental health (related to risk of mental disorders). These characteristics are relevant to the research, as they can protect or affect levels of resilience [33,34,35]. In addition, working time may be related to the aforementioned variables, which is not yet clear as no studies have been found to date.

Amongst military personnel, time in service may be a protective factor against mental disorders (e.g., major depressive disorder and post-traumatic stress disorder [PTSD]) as it may be mediated by the development of resilience. However, there is little evidence on the influence of work time on resilience in this population, even less so in those who have been the first line of defense against the COVID-19 pandemic. A pre-pandemic study of U.S. military veterans showed that the more years of service, the higher the resilience score [36]. A secondary analysis of the Millennium Cohort, a prospective population-based study of U.S. military personnel prior to operations in Iraq and Afghanistan in 2001, showed that nearly 8 out of 10 military personnel maintained a consistent level of resilience seven years after their military deployment [37]. Another descriptive study of Australian servicemen also reported that the majority exhibited resilient characteristics four years after the start of military service [38].

In addition, previous studies that have described our association of interest have some methodological limitations, such as small sample size [36], the non-inclusion of service time rendered by military personnel to their institution [36] and selection bias [39]. The development of resilience through years of service has been previously demonstrated in the military population during the pre-pandemic period [36], and suggested as a protective factor against PTSD [37,38]. Therefore, this study aimed to assess whether work time is associated with resilience using data from a previous survey of front-line military personnel during the second epidemic wave of COVID-19. We hypothesized that the more months the military served, the greater the capacity to adapt to the adversity of the health emergency, which would be reflected in higher scores on the resilience CD-RISC scale. In contrast, those military recruited more recently (e.g., in the second wave) would not show an adequate resilience pattern. Additionally, the prevalence of resilience levels and the frequency of each item included in the CD-RISC were estimated.

## 2. Materials and Methods

### 2.1. Study Design

A secondary data analysis was performed to evaluate the association between working time and resilience. The primary study had a cross-sectional design and was conducted amongst the military personnel of Lambayeque, Peru from 2 to 9 November 2021, during the second epidemic wave in this region.

### 2.2. Population and Sample

The population consisted of 820 military personnel working on the first line of defense against COVID-19 in Lambayeque, Peru. The members were part of different units (e.g., operational, administrative and support). In the primary study, we estimated a required sample size of 582 military personnel, after using the following data: 12.8% expected prevalence of PTSD (the outcome in the primary study) [40], 99% confidence level, 2.5% precision, and 20% loss-rejection rate of participants. Finally, we were able to enroll a larger sample size than estimated (*n* = 710). Participants gave their consent to participate and at the time of their participation were actively working on the front line of defense in the health emergency. For this secondary data analysis, we excluded 124 military personnel who did not complete the short form of the CD-RISC, since it measured the outcome of interest (resilience). Therefore, we used a sample of 586 participants. Sampling for the primary study was nonrandom.

### 2.3. Measures

The outcome was resilience, defined as a score of more than 30 points obtained from the sum of answers to questions on the CD-RISC. This cut-off point was previously applied in a Peruvian study [41] based on Spanish validation studies [42,43]. The instrument is composed of 10 questions that measure resilience. It presents adequate reliability and validity properties [44]. An optimal global internal consistency has been estimated (Cronbach’s alpha: 0.85) [44]. It uses a Likert scale from 0 “not at all”, 1 “rarely”, 2 “sometimes”, 3 “often”, to 4 “almost always”. It has been validated in Latin American population [45,46,47,48] and used in a veteran military population [25]. In the present study, the short form of the CD-RISC showed good overall internal consistency (Cronbach’s alpha: 0.972).

The exposure was working time, defined as the reported number of months that a military member has worked in the COVID-19 first line of defense activities since the beginning of the pandemic health emergency. This variable was categorized into 4 values: 1 to 6 months, 7 to 12 months, 13 to 18 months, and more than 18 months. Since resilience is a characteristic that appears to develop over time in the military population [36], we have decided to categorize how resilience varies in each period so that appropriate measures could be provided according to each period of exposure.

Secondary variables were age (in years), sex (male, female), marital status (single, married, cohabiting, divorced), religion (none, Catholic, non-Catholic), parenting (no, yes), report of frequent alcohol consumption (no, yes), report of frequent tobacco consumption (no, yes), reported weight (kg) and height (cm) with which body mass index was estimated and categorized as normal, overweight, and obese; self-report of personal and family history of mental health problems (no, yes), self-report of seeking mental health help during the pandemic (no, yes), and self-report of trust in the government to handle the pandemic (no, yes).

### 2.4. Procedures

We used REDCap (Research Electronic Data Capture) 8.1.8 2022 Vanderbilt University (Nashville, TN, USA) to design the questionnaires of interest, create a study dissemination link and ensure quality control of the data collected. After obtaining authorization from the ethics committee and military authority of the study site, the research team coordinated the face-to-face execution of the interviews. The interviews were scheduled in the morning and afternoon for one week, ensuring compliance with biosecurity measures at all times. The research team disseminated the link to the military supervisor, who sent the link to his staff through internal work groups. First, informed consent was requested electronically, which was inserted in the first part of the disseminated link. The research team resolved the doubts of participants that were generated at the time of completing the survey, such as problems entering the link, unanswered questions that generated a warning message and prevented the survey from continuing, and questions that participants did not understand.

### 2.5. Data Analysis

Statistical analysis was performed in Stata v.17.0 (StataCorp LP, College Station, TX, USA).

For descriptive analysis of categorical variables, absolute frequencies and percentages were reported. For numerical variables, the assumption of normal distribution was evaluated and then the best measure of central tendency and dispersion was reported.

For the bivariate analysis, the association between resilience and working time and other covariates was assessed using the chi-square test (for categorical variables) after evaluating the expected frequency assumption, or the Mann–Whitney U-test (for numerical variables) after evaluation of the nonnormal data distribution.

Multivariate analysis was performed using generalized linear models with a Poisson distribution family, log link function, and robust variance. Simple regression models were used to assess the unadjusted association between resilience and working time, as well as the association with the rest of the covariates. Multiple regression analysis was then applied to identify whether and how the confounders modified the magnitude and direction of association between working time and resilience. Prevalence ratios (PR) and 95% confidence intervals were estimated. Collinearity was evaluated between the variables of interest.

## 3. Results

Of 586 participants, 94% were male, 72.9% were single, and 70% reported being Catholic; 33.3% were overweight and 1.2% reported a prior history of mental health problem. Over half of them (53.9%) reported confidence in the government to manage the COVID-19 pandemic, while 36.3% were working in the first line of defense against COVID-19 for more than 18 months. The mean score for resilience was 22.18 points and 43.2% had a high level of resilience (Table 1).

**Figure 1:**
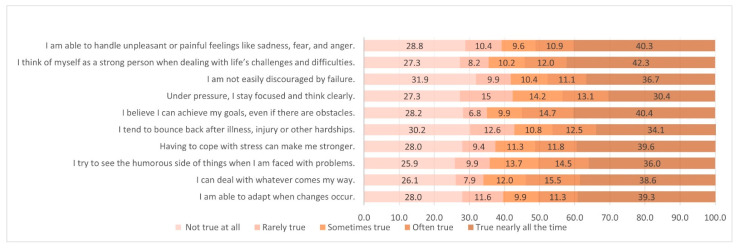
Distribution of responses from the short form of the CD-RISC.

The responses to the CD-RISC items are shown in Figure 1. Among the respondents, 42.3% reported that they almost always believe they are a strong person when facing challenges and difficulties, 40.3% mentioned that they are able to handle unpleasant feelings, and 40.4% reported that they almost always achieve their goals despite obstacles.

Military personnel with more than 18 months working in the first line of defense against COVID-19 had a 12.7% higher frequency of high level resilience, compared with military personnel whose working time was in the range 1 to 6 months (51.9% vs. 39.2%; *p* = 0.022). The remaining covariates were not statistically associated with resilience (Table 2).

The results from the regression analyses are shown in Table 3. The simple regression model showed that the frequency of high level of resilience increased by 32% in military personnel with more than 18 months of working time as the first line of defense against COVID-19, compared with military personnel with less than 7 months of working time (PR: 1.06; 95% CI: 0.77–1.45). This association was maintained in terms of direction and magnitude in the multiple regression model: military members with more than 18 months of working time had 35% higher frequency of high level resilience (PR: 1.35; 95% CI: 1.05–1.75). Additionally, cohabitants had 73% higher prevalence of high level resilience (PR: 1.73; 95% CI: 1.15–2.61).

## 4. Discussion

### 4.1. Prevalence of Resilience in the Military

We found that 43.2% of the military presented a high level of resilience and the mean resilience score was 22.18 points, according to the 10-item CD-RISC. In addition, according to the items of this scale, it was observed that approximately four out of ten military personnel reported that (a) they are able to adapt when changes arise, (b) difficulties can make them stronger, (c) they are strong individuals when faced with challenges or difficulties, and (d) they tend to recover soon after difficulties.

The scores of the CD-RISC items are similar to the results of a North American study in the pre-pandemic period, in which the mean resilience score was found to be high (scores greater than 3 points on a total 4-point scale), using the same short form version of the instrument [21]. In addition, the overall mean score found in this research is consistent with that of a study conducted in the Irish military, in which the short form of the CD-RISC was also used (mean score 29.68 points) [24]. However, it is relatively lower than in a study of U.S. Air Force personnel during the pandemic, in which the mean resilience score was found to be higher than the 75th percentile [20]. It is also lower than that reported in a pre-pandemic study of U.S. veterans, in which 75% of military personnel were found to have a high level of resilience using the 5-item CD-RISC [22]. Another study in veterans from the same country showed that the mean resilience score was above the 50th percentile according to the 25-item CD-RISC [23]. A study of U.S. military personnel, also using the 25-item CD-RISC, showed a high mean score for resilience [21], and a Swiss study found mean resilience close to the 75th percentile [25]. In Colombia, it was observed that military personnel showed a mean score of 29 points using the short form of the CD-RISC [26], while in Peru, a study in the VRAEM military showed a resilience frequency of 60% using the Wagnild and Young resilience scale [27]. The mean score of resilience is lower in the present study probably because it was conducted during the pandemic, whereas most of the previous studies were conducted before the health emergency. In addition, this result is hardly comparable with those in the literature because different versions of the CD-RISC were used and the target population was mostly veterans. Military training includes intense mental preparation to handle extreme emotional circumstances such as war or national defense events [49]. Knowing the prevalence of resilience in this study allows us to understand that military personnel might not be adequately trained to face events such as the pandemic and provide support in different areas, such as health, transportation and security. It is important that measures are taken to improve resilience values in the Peruvian military population.

### 4.2. Working Time and Resilience during the Pandemic

In the present investigation, it was found that having more than 18 months working in the first line of defense against the COVID-19 pandemic increased the frequency of high level resilience by 32%. A pre-pandemic study in veterans showed a similar association in which the more years of service, the higher the resilience score, according to the RS-14 resilience scale [36]. In contrast, a secondary analysis of the Millennium Cohort, a prospective population-based study of U.S. military personnel prior to operations in Iraq and Afghanistan in 2001, showed that nearly 8 out of 10 military personnel maintained a stable score (around 20 points) on the PCL-C scale, which translates as a consistent level of resilience seven years after their military deployment [37]. Another descriptive study in Australian servicemen also reported that the majority exhibited resilient characteristics, and the mean score on the PCL-4 remained at a minimum (around 4 points out of 20) four years after the start of military service [38]. In addition, those who started with higher PTSD symptom severity and then experienced a considerable decrease in PCL-4 score (from 8 to 6 points out of 20) showed a higher frequency of self-blaming (OR = 2.09; 95% CI = 1.91–2.29), risk-taking (OR = 1.97; 95% CI = 1.70–2.29), and avoidance (OR = 1.79; 95% CI = 1.55–2.06) coping styles [38]. This improvement in PTSD symptoms could be attributed to the progressive development of resilience skills. Likewise, the present result differs from that reported in a study of US military personnel, in which it was found that despite a reported high level of resilience at the beginning of a military operation, this protective factor decreased over time as an increase in scores on the adapted CIDI-SC scale to measure anger symptoms was observed [50].

These studies in general support our hypothesis that time in service increases the levels of resilience during the pandemic amongst military personnel. The association found in the present study could be explained by some particular situations. First, the experience of the first wave would have generated an adaptation that would have improved the levels of resilience observed in this study (conducted in the second wave). In addition, the high levels of resilience could also have been the result of the training and experience of some military personnel to face war events, which makes them more resilient to critical situations such as the pandemic [51]. This resilience has developed particularly in an environment in which there was a high incidence of COVID-19 cases [52]. The present results suggest that prolonged exposure to the pandemic-like coping environment has a benefit for the mental health of military members.

Time plays an important role in the development of resilience. Adverse events force an individual to adapt, but some develop mental health problems (e.g., major depressive disorders and PTSD) while others develop resilience [53]. In this process, neurochemical, genetic, and epigenetic processes determine over time how to react against stressful events [54]. Individual resilience profiles can help in the prevention of mental disorders. However, the development of resilience in military personnel has been shown to be based on mindfulness and purpose in life [13,20], suggesting that they rely on a period of reflection rather than immediate response.

### 4.3. Implications of Findings for Mental Health Policy

The results of this study show that resilience in the military is essential for handling emotionally charged situations. The development of this skill is complex, but evidence supports that this relies on a period of adaptation. For mental health policy, identifying which military members are more susceptible to mental health problems during their early period of service can help focus strategies on prevention and early treatment. This should be recognized as a public health priority, as these personnel provide important support in emergency situations to maintain safety and order [55,56]. It is also important to improve resilience indicators in the military population as those with low resilience are exposed to greater negative mental health outcomes than the general population. The findings in the present study are aimed at health authorities working in the military. They may also serve for comparison with other Peruvian [27] and Latin American studies [26].

### 4.4. Limitations and Strengths

The study has several limitations. First, the study has a cross-sectional design so that causality between the associated variables cannot be established. Secondly, the assessment of variables by survey may introduce recall bias or the intention to conceal the actual response. Specifically, mental health-related variables such as the personal mental health history question may generate a social acceptability bias. Third, the study may present information bias due to the absence of some relevant confounders that were not part of the primary study, such as the presence of coping styles, number of traumatic events, and social support [38], which could modify the association found. Fourth, the study is specific to a Peruvian military population, so the results cannot be extrapolated to the entire Peruvian population. Fifth, there is selection bias since the study was conducted by non-probabilistic sampling, and the results cannot be extrapolated to the entire study population. However, this research is one of the few that evaluates the importance of working time in the development of resilience in a region strongly affected by the pandemic [52]. In this sense, the high levels of resilience of Lambayeque’s military personnel in this context are also evident. Finally, it has evaluated a considerable number of military personnel who have worked on the front line of defense against the COVID-19, and to our knowledge it is the first documented evaluation to report on resilience and potential factors influencing it.

## 5. Conclusions

It was found that 4 out of 10 military personnel presented a high level of resilience. In addition, working time greater than 18 months was associated with a high level of resilience. These findings suggest that Peruvian military personnel have acquired a strong mental preparedness to face critical situations during the pandemic. It also reinforces the idea that prolonged exposure to the military environment strengthens resilience and protects against potential emotional disturbances. Further studies are needed to deepen the knowledge of the mental state of the military population and its association with service time and other relevant factors in order to improve mental health prevention strategies.

## Figures and Tables

**Table 1:** Characteristics of participants (*n* = 586).

Characteristics		*n* (%)
Age (years) *		22 (19–32)
Sex		
	Female	35 (6.0)
	Male	551 (94.0)
Marital status		
	Single	427 (72.9)
	Married	138 (23.6)
	Cohabitant	13 (2.2)
	Divorced	8 (1.4)
Religion		
	None	83 (14.2)
	Catholic	410 (70.0)
	Non-Catholic	93 (15.9)
Parenting (yes)		161 (27.5)
Alcoholism (yes)		99 (17.0)
Smoking (yes)		38 (6.5)
Comorbidity		
	Hypertension	55 (9.4)
	Diabetes	11 (1.9)
BMI		
	Normal	346 (60.0)
	Overweight	192 (33.3)
	Obesity	39 (6.8)
Personal history of mental health (yes)		7 (1.2)
Family history of mental health (yes)		25 (4.3)
Seeking mental health help (yes)		47 (8.0)
Confidence in government to manage COVID-19		
	Yes	316 (53.9)
	No	270 (46.1)
Working time		
	1 to 6 months	148 (25.8)
	7 to 12 months	94 (16.4)
	13 to 18 months	123 (21.5)
	19 months and over	208 (36.3)
Resilience **		22.18 ± 14.96
Resilience (categorized)		
	Low	333 (56.8)
	High	253 (43.2)

* Median (25–75th percentile); ** Mean ± standard deviation.

**Table 2:** Characteristics associated with resilience in bivariate analysis.

Variables		Resilience		*p* *
		Low (*n* = 333)	High (*n* = 253)	
		*n* (%)	*n* (%)	
Age (years) **		22 (19–32)	22 (19–32)	0.681 ***
Sex				0.696
	Female	21 (60.0)	14 (40.0)	
	Male	312 (56.6)	239 (43.4)	
Marital status				0.583
	Single	245 (57.4)	182 (42.6)	
	Married	78 (56.5)	60 (43.5)	
	Cohabitant	5 (38.5)	8 (61.5)	
	Divorced	5 (62.5)	3 (37.5)	
Religion				0.181
	None	53 (63.9)	30 (36.1)	
	Catholic	223 (54.4)	187 (45.6)	
	Non-Catholic	57 (61.3)	36 (38.7)	
Parenting (yes)		92 (57.1)	69 (42.9)	0.924
Alcoholism (yes)		57 (57.6)	42 (42.4)	0.869
Smoking (yes)		27 (71.1)	11 (29.0)	0.067
Comorbidity				
	Hypertension	35 (63.6)	20 (36.4)	0.284
	Diabetes	7 (63.6)	4 (36.4)	0.645
BMI				0.095
	Normal	200 (57.8)	146 (42.2)	
	Overweight	99 (51.6)	93 (48.4)	
	Obesity	27 (69.2)	12 (30.8)	
Personal history of mental health				0.121
	No	327 (56.5)	252 (43.5)	
	Yes	6 (85.7)	1 (14.3)	
Family history of mental health				0.117
	No	315 (56.2)	246 (43.9)	
	Yes	18 (72.0)	7 (28.0)	
Seeking mental health help				0.828
	No	307 (57.0)	232 (43.0)	
	Yes	26 (55.3)	21 (44.7)	
Confidence in government to manage COVID-19				0.684
	Yes	182 (57.6)	134 (42.4)	
	No	151 (55.9)	119 (44.1)	
Working time				**0.022**
	1 to 6 months	90 (60.8)	58 (39.2)	
	7 to 12 months	55 (58.5)	39 (41.5)	
	13 to 18 months	78 (63.4)	45 (36.6)	
	19 months and over	100 (48.1)	108 (51.9)	

* *p*-Value of categorical variables calculated with the chi-square test; ** Median-interquartile range; *** *p*-Value calculated with the Mann–Whitney U-test.

**Table 3:** Factors associated with resilience in simple and multiple regression analysis.

Characteristics		Resilience					
		Simple Regression			Multiple Regression		
		PR	95% CI	*p* *	PR	95% CI	*p* *
Age (years)		1.00	0.99–1.01	0.932	0.99	0.97–1.01	0.279
Sex							
	Female	Ref.			Ref.		
	Male	1.08	0.71–1.65	0.703	1.14	0.74–1.75	0.549
Marital status							
	Single	Ref.			Ref.		
	Married	1.02	0.82–1.27	0.859	1.08	0.71–1.64	0.733
	Cohabitant	1.44	0.93–2.25	0.105	1.73	1.15–2.61	**0.009**
	Divorced	0.88	0.36–2.17	0.781	1.09	0.43–2.76	0.855
Religion							
	None	Ref.			Ref.		
	Catholic	1.26	0.93–1.71	0.135	1.28	0.95–1.73	0.107
	Non-Catholic	1.07	0.73–1.57	0.726	1.16	0.80–1.70	0.425
Parenting (yes)		0.99	0.80–1.22	0.924	0.90	0.62–1.29	0.563
Alcoholism (yes)		0.98	0.76–1.26	0.870	1.05	0.80–1.36	0.741
Smoking (yes)		0.66	0.36–1.20	0.171	0.68	0.41–1.12	0.131
Comorbidity							
	Hypertension	0.83	0.58–1.19	0.310	0.92	0.64–1.32	0.635
	Diabetes	0.84	0.38–1.85	0.664	1.32	0.62–2.82	0.472
BMI							
	Normal	Ref.			Ref.		
	Overweight	1.15	0.95–1.39	0.157	1.11	0.90–1.38	0.336
	Obesity	0.73	0.45–1.19	0.204	0.72	0.44–1.17	0.182
Personal history of mental health							
	No	Ref.			Ref.		
	Yes	0.33	0.05–2.02	0.230	0.36	0.07–1.97	0.238
Family history of mental health							
	No	Ref.			Ref.		
	Yes	0.64	0.34–1.21	0.167	0.75	0.41–1.36	0.338
Seeking mental health help							
	No	Ref.			Ref.		
	Yes	1.04	0.74–1.45	0.826	1.16	0.84–1.61	0.364
Confidence in government to manage COVID-19							
	Yes	Ref.			Ref.		
	No	1.04	0.86–1.25	0.684	1.07	0.88–1.29	0.516
Working time							
	1 to 6 months	Ref.			Ref.		
	7 to 12 months	1.06	0.77–1.45	0.721	1.04	0.76–1.42	0.798
	13 to 18 months	0.93	0.69–1.27	0.661	0.97	0.71–1.32	0.842
	19 months and over	1.32	1.04–1.68	**0.021**	1.35	1.05–1.75	**0.021**

* *p*-Values obtained with Generalized Linear Models (GLM), Poisson family, log-link function, and robust variance.

## Data Availability

The dataset generated and analyzed during the current study is not publicly available because the ethics committee has not provided permission/authorization to publicly share the data but are available from the corresponding author on reasonable request.

## References

[1] Sisto A., Vicinanza F., Campanozzi L.L., Ricci G., Tartaglini D., Tambone V. (2019). Towards a Transversal Definition of Psychological Resilience: A Literature Review. Medicina.

[2] Quesada C.V. (2003). El concepto de resiliencia individual y familiar. Aplicaciones en la intervención social. Psychosoc. Interv..

[3] Chang J., Yuan Y., Wang D. (2020). Mental health status and its influencing factors among college students during the epidemic of COVID-19. Nan Fang Yi Ke Da Xue Xue Bao.

[4] Wang X., Hegde S., Son C., Keller B., Smith A., Sasangohar F. (2020). Investigating Mental Health of US College Students during the COVID-19 Pandemic: Cross-Sectional Survey Study. J. Med. Internet Res..

[5] Ponce V.V., Garrido M.V., Peralta C.I., Astudillo D., Malca J.T., Manrique E.O., Quispe E.T. (2020). Factores Asociados al Afrontamiento Psicológico Frente a La COVID-19 Durante El Periodo de Cuarentena. Rev. Cuba. Med. Mil..

[6] Gloster A.T., Lamnisos D., Lubenko J., Presti G., Squatrito V., Constantinou M., Nicolaou C., Papacostas S., Aydın G., Chong Y.Y. (2020). Impact of COVID-19 Pandemic on Mental Health: An International Study. PLoS ONE.

[7] Jones E.A.K., Mitra A.K., Bhuiyan A.R. (2021). Impact of COVID-19 on Mental Health in Adolescents: A Systematic Review. Int. J. Environ. Res. Public Health.

[8] Buselli R., Corsi M., Baldanzi S., Chiumiento M., Del Lupo E., Dell’Oste V., Bertelloni C.A., Massimetti G., Dell’Osso L., Cristaudo A. (2020). Professional Quality of Life and Mental Health Outcomes among Health Care Workers Exposed to SARS-CoV-2 (COVID-19). Int. J. Environ. Res. Public Health.

[9] Pappa S., Ntella V., Giannakas T., Giannakoulis V.G., Papoutsi E., Katsaounou P. (2020). Prevalence of Depression, Anxiety, and Insomnia among Healthcare Workers during the COVID-19 Pandemic: A Systematic Review and Meta-Analysis. Brain Behav. Immun..

[10] Na P., Tsai J., Harpaz-Rotem I., Pietrzak R. (2022). Mental Health and Suicidal Ideation in US Military Veterans with Histories of COVID-19 Infection. BMJ Mil. Health.

[11] Gamboa-Moreno L.N., Becerra-Rodríguez K.G., Lopez-Vergara Y.I., Goicochea-Ríos E., Gamboa-Moreno L.N., Becerra-Rodríguez K.G., Lopez-Vergara Y.I., Goicochea-Ríos E. (2021). Nivel de Resiliencia Del Personal de Salud Frente a La Pandemia Por COVID-19. Rev. Cuerpo Médico Hosp. Nac. Almanzor Aguinaga Asenjo.

[12] Killgore W.D.S., Taylor E.C., Cloonan S.A., Dailey N.S. (2020). Psychological Resilience during the COVID-19 Lockdown. Psychiatry Res..

[13] Kachadourian L., Tsai J., Na P.J., Krystal J.H., Southwick S.M., Pietrzak R.H. (2021). Resilience in the Face of the COVID-19 Pandemic: A National Study of US Military Veterans. Prim. Care Companion CNS Disord..

[14] Garayar-Peceros H., Prado-Martínez F., Cortez-Soto A.G., de Guzmán S.N., García-Gutiérrez J.G., Alarco J.J. (2021). Actitudes hacia la pandemia y su relación con la resiliencia en estudiantes de medicina peruanos. Inv. Ed. Med..

[15] Timalsina R., Songwathana P., Sae-Sia W. (2021). Resilience and Its Associated Factors among Older Disaster Survivors. Geriatr Nurs.

[16] Olaimat A.N., Aolymat I., Elsahoryi N., Shahbaz H.M., Holley R.A. (2020). Attitudes, Anxiety, and Behavioral Practices Regarding COVID-19 among University Students in Jordan: A Cross-Sectional Study. Am. J. Trop. Med. Hyg..

[17] Peng Y., Pei C., Zheng Y., Wang J., Zhang K., Zheng Z., Zhu P. (2020). A Cross-Sectional Survey of Knowledge, Attitude and Practice Associated with COVID-19 among Undergraduate Students in China. BMC Public Health.

[18] Ikhlaq A., Bint-E-Riaz H., Bashir I., Ijaz F. (2020). Awareness and Attitude of Undergraduate Medical Students towards 2019-Novel Corona Virus. Pak. J. Med. Sci..

[19] Van der Merwe L.J., Botha A., Joubert G. (2020). Resilience and Coping Strategies of Undergraduate Medical Students at the University of the Free State. S. Afr. J. Psychiatr..

[20] Ligeza N., Larson A., DeBeliso M. (2022). Resilience, Psychological Stress, Physical Activity, and BMI among United States Air National Guardsmen: The COVID-19 Pandemic. J. Lifestyle Med..

[21] Bezdjian S., Schneider K.G., Burchett D., Baker M.T., Garb H.N. (2017). Resilience in the United States Air Force: Psychometric Properties of the Connor-Davidson Resilience Scale (CD-RISC). Psychol. Assess..

[22] Adams R.E., Hu Y., Figley C.R., Urosevich T.G., Hoffman S.N., Kirchner H.L., Dugan R.J., Boscarino J.J., Withey C.A., Boscarino J.A. (2021). Risk and Protective Factors Associated with Mental Health among Female Military Veterans: Results from the Veterans’ Health Study. BMC Womens Health.

[23] Rakesh G., Clausen A.N., Buckley M.N., Clarke-Rubright E., Fairbank J.A., Wagner H.R., Morey R.A. (2022). The Role of Trauma, Social Support, and Demography on Veteran Resilience. Eur. J. Psychotraumatol..

[24] Mitchell N.A., McCauley M., O’Brien D., Wilson C.E. (2022). Mental Health and Resilience in the Irish Defense Forces during the COVID-19 Global Pandemic. Mil. Psychol..

[25] Green K.T., Hayward L.C., Williams A.M., Dennis P.A., Bryan B.C., Taber K.H., Davidson J.R.T., Beckham J.C., Calhoun P.S., Ingle S.J. (2014). Examining the Factor Structure of the Connor-Davidson Resilience Scale (CD-RISC) in a Post-9/11 U.S. Military Veteran Sample. Assessment.

[26] Herrera-Moreno D., Carvajal-Ovalle D., Cueva-Nuñez M.A., Acevedo C., Riveros-Munévar F., Camacho K., Fajardo-Tejada D.M., Clavijo-Moreno M.N., Lara-Correa D.L., Vinaccia-Alpi S. (2018). Body Image, Perceived Stress, and Resilience in Military Amputees of the Internal Armed Conflict in Colombia. Int. J. Psychol. Res..

[27] Huanay Yauli M.M. (2019). Resiliencia y estilos de afrontamiento al estrés en soldados que prestan servicio militar voluntario en zona VRAEM del Ejército Peruano. Repositorio Institucional-Continental.

[28] Labrague L.J. (2021). Psychological Resilience, Coping Behaviours and Social Support among Health Care Workers during the COVID-19 Pandemic: A Systematic Review of Quantitative Studies. J. Nurs. Manag..

[29] Mosheva M., Hertz-Palmor N., Ilan S.D., Matalon N., Pessach I.M., Afek A., Ziv A., Kreiss Y., Gross R., Gothelf D. (2020). Anxiety, Pandemic-Related Stress and Resilience among Physicians during the COVID-19 Pandemic. Depress. Anxiety.

[30] Baskin R.G., Bartlett R. (2021). Healthcare Worker Resilience during the COVID-19 Pandemic: An Integrative Review. J. Nurs. Manag..

[31] Amieva H., Avila-Funes J.-A., Caillot-Ranjeva S., Dartigues J.-F., Koleck M., Letenneur L., Pech M., Pérès K., Raoux N., Rascle N. (2021). Older People Facing the Crisis of COVID-19: Between Fragility and Resilience. J. Frailty Aging.

[32] Croghan I.T., Chesak S.S., Adusumalli J., Fischer K.M., Beck E.W., Patel S.R., Ghosh K., Schroeder D.R., Bhagra A. (2021). Stress, Resilience, and Coping of Healthcare Workers during the COVID-19 Pandemic. J. Prim. Care Community Health.

[33] Chang M.-C., Chen P.-F., Lee T.-H., Lin C.-C., Chiang K.-T., Tsai M.-F., Kuo H.-F., Lung F.-W. (2021). The Effect of Religion on Psychological Resilience in Healthcare Workers during the Coronavirus Disease 2019 Pandemic. Front. Psychol..

[34] Dullius A.A.d.S., Fava S.M.C.L., Ribeiro P.M., Terra F.d.S. (2018). Alcohol Consumption/Dependence and Resilience in Older Adults with High Blood Pressure 1. Rev. Lat. Am. Enferm..

[35] Wu Y., Sang Z., Zhang X.-C., Margraf J. (2020). The Relationship between Resilience and Mental Health in Chinese College Students: A Longitudinal Cross-Lagged Analysis. Front. Psychol..

[36] Rice V., Liu B. (2016). Personal Resilience and Coping Part II: Identifying Resilience and Coping among U.S. Military Service Members and Veterans with Implications for Work. Work.

[37] Bonanno G.A., Mancini A.D., Horton J.L., Powell T.M., Leardmann C.A., Boyko E.J., Wells T.S., Hooper T.I., Gackstetter G.D., Smith T.C. (2012). Trajectories of Trauma Symptoms and Resilience in Deployed U.S. Military Service Members: Prospective Cohort Study. Br. J. Psychiatry.

[38] Dell L., Casetta C., Benassi H., Cowlishaw S., Agathos J., O’Donnell M., Crane M., Lewis V., Pacella B., Terhaag S. (2022). Mental Health across the Early Years in the Military. Psychol. Med..

[39] Sefidan S., Pramstaller M., La Marca R., Wyss T., Sadeghi-Bahmani D., Annen H., Brand S. (2021). Resilience as a Protective Factor in Basic Military Training, a Longitudinal Study of the Swiss Armed Forces. Int. J. Environ. Res. Public Health.

[40] Pietrzak R.H., Tsai J., Southwick S.M. (2021). Association of Symptoms of Posttraumatic Stress Disorder With Posttraumatic Psychological Growth Among US Veterans During the COVID-19 Pandemic. JAMA Netw. Open.

[41] Leiva León N.F. (2021). La Resilencia Como Factor Asociado Al Sindrome de Burnout, Depresión y Ansiedad en El Personal de Salud Que Labora en Las Unidades de Cuidados Intensivos Durante la Pandemia COVID-19 en El Perú. https://hdl.handle.net/20.500.12866/8964.

[42] Sánchez M.I.S., de Pedro M.M., Izquierdo M.G. (2016). Propiedades psicométricas de la versión española de la escala de resiliencia de 10 ítems de Connor-Davidson (CD-RISC 10) en una muestra multiocupacional. Rev. Latinoam. De Psicol..

[43] Propiedades Psicométricas de la Versión Española de la Escala de Resiliencia de 10 ítems de Connor-Davidson (CD-RISC 10) en una Muestra de Desempleados|Summa Psicológica. https://dialnet.unirioja.es/servlet/articulo?codigo=7009141.

[44] Campbell-Sills L., Stein M.B. (2007). Psychometric Analysis and Refinement of the Connor-Davidson Resilience Scale (CD-RISC): Validation of a 10-Item Measure of Resilience. J. Trauma. Stress.

[45] Blanco V., Guisande M.A., Sánchez M.T., Otero P., Vázquez F.L. (2019). Spanish Validation of the 10-Item Connor-Davidson Resilience Scale (CD-RISC 10) with Non-Professional Caregivers. Aging Ment. Health.

[46] Serrano-Parra M.D., Abejar M.G., Notario-Pacheco B., Bartolomé-Gutiérrez R., Solera-Martínez M., Vizcaino V.M. (2013). Validity of the Connor-Davidson resilience scale (10 items) in a population of elderly. Enferm. Clin..

[47] Notario-Pacheco B., Martínez-Vizcaíno V., Trillo-Calvo E., Pérez-Yus M.C., Serrano-Parra D., García-Campayo J. (2014). Validity and Reliability of the Spanish Version of the 10-Item CD-RISC in Patients with Fibromyalgia. Health Qual. Life Outcomes.

[48] Notario-Pacheco B., Solera-Martínez M., Serrano-Parra M.D., Bartolomé-Gutiérrez R., García-Campayo J., Martínez-Vizcaíno V. (2011). Reliability and Validity of the Spanish Version of the 10-Item Connor-Davidson Resilience Scale (10-Item CD-RISC) in Young Adults. Health Qual. Life Outcomes.

[49] Guo R., Sun M., Zhang C., Fan Z., Liu Z., Tao H. (2021). The Role of Military Training in Improving Psychological Resilience and Reducing Depression among College Freshmen. Front. Psychiatry.

[50] Campbell-Sills L., Kautz J.D., Choi K.W., Naifeh J.A., Aliaga P.A., Jain S., Sun X., Kessler R.C., Stein M.B., Ursano R.J. (2021). Effects of Prior Deployments and Perceived Resilience on Anger Trajectories of Combat-Deployed Soldiers. Psychol. Med..

[51] Marini C.M., Pless Kaiser A., Smith B.N., Fiori K.L. (2020). Aging Veterans’ Mental Health and Well-Being in the Context of COVID-19: The Importance of Social Ties during Physical Distancing. Psychol. Trauma..

[52] Díaz-Vélez C., Failoc-Rojas V.E., Valladares-Garrido M.J., Colchado J., Carrera-Acosta L., Becerra M., Paico D.M., Ocampo-Salazar E.T. (2021). SARS-CoV-2 Seroprevalence Study in Lambayeque, Peru. June–July 2020. PeerJ.

[53] Boyce W.T., Levitt P., Martinez F.D., McEwen B.S., Shonkoff J.P. (2021). Genes, Environments, and Time: The Biology of Adversity and Resilience. Pediatrics.

[54] Osório C., Probert T., Jones E., Young A.H., Robbins I. (2017). Adapting to Stress: Understanding the Neurobiology of Resilience. Behav. Med..

[55] Gibson-Fall F. (2021). Military Responses to COVID-19, Emerging Trends in Global Civil-Military Engagements. Rev. Int. Stud..

[56] Michaud J., Moss K., Licina D., Waldman R., Kamradt-Scott A., Bartee M., Lim M., Williamson J., Burkle F., Polyak C.S. (2019). Militaries and Global Health: Peace, Conflict, and Disaster Response. Lancet.

